# Evaluation and Validation of the Roche Elecsys SARS-CoV-2 Antigen Electro-Chemiluminescent Immunoassay in a Southeast Asian Region

**DOI:** 10.3390/vaccines10020198

**Published:** 2022-01-27

**Authors:** Chin Shern Lau, Soon Kieng Phua, See Ping Hoo, Boran Jiang, Tar-Choon Aw

**Affiliations:** 1 Department of Laboratory Medicine, Changi General Hospital, Singapore 529889, Singapore; soon_kieng_phua@cgh.com.sg (S.K.P.); See_Ping_Hoo@cgh.com.sg (S.P.H.); jiang_boran@cgh.com.sg (B.J.); aw.tar.choon@singhealth.com.sg (T.-C.A.); 2 Department of Microbiology, Changi General Hospital, Singapore 529889, Singapore; 3 Department of Medicine, National University of Singapore, Singapore 119077, Singapore; 4 Academic Pathology Program, Duke-NUS Medical School, Singapore 169857, Singapore

**Keywords:** COVID-19, SARS-CoV-2, antigen, RT-PCR, central laboratory testing

## Abstract

Introduction: SARS-CoV-2 antigen tests can complement and substitute for RT-PCR tests. Centralized laboratory automated SARS-CoV-2 antigen tests that can be scaled to process a large number COVID-19 cases simultaneously are now available. We have evaluated the new Roche Elecsys SARS-CoV-2 antigen electro-chemiluminescent immunoassay. Methods: The Roche SARS-CoV-2 antigen assay is a double-antibody sandwich electro-chemiluminescent immunoassay, which reports a cut-off index (COI) (COI ≥ 1.0 considered positive). We assessed assay precision and linearity, and confirmed the reactivity limit. We determined the assay sensitivity and specificity with a verification group (289 controls and 61 RT-PCR positive COVID-19 cases). Assay performance was also validated against the consecutive samples we received (7657 controls and 17 cases) for SARS-CoV-2 antigen testing from June to October 2021. Result: The assay had a within-run precision CV of 3.0% at COI 0.68, and a CV of 1.5% at COI 3.49. Between-run precision was 3.0% at COI 0.68 and 1.8% at COI 3.49. The assay was linear from COI 0.65 to 7.84. All 35 C_50_ ± 20% test results performed over 7 days were positive/negative, respectively. In the verification group, overall sensitivity was 42.6% (26/61 positive, 95% CI 30.0–55.9), and specificity was 99.7% (1/289 positive, 95% CI 98.1–100). The agreement between the SARS-CoV-2 antigen and the RT-PCR cycle threshold (Ct) count was good (r = 0.90). In cases with Ct counts ≤ 30, the antigen assay sensitivity improved to 94.7% (18/19 positive, 95% CI 74.0–99.9). In our validation group, antigen sensitivity was 62.5% (5/8 antigen positive, 95% CI 24.5–91.5) within the first week of disease onset, but no cases were reactive after the first week of disease onset. Conclusion: The Elecsys SARS-CoV-2 antigen assay has good performance within manufacturer specifications. The sensitivity of the Roche antigen assay was greatest when used in patients with lower RT-PCR Ct values (≤30) and within the first week of disease onset.

## 1. Introduction

The WHO recommends that confirmation of cases with COVID-19 be based on the detection of the specific SARS-CoV-2 gene target(s) using reverse transcription–polymerase chain reaction (RT-PCR) testing [1]. However, RT-PCR testing capabilities are costly, and can be overwhelmed by the large volume of samples collected for SARS-CoV-2 testing in population screening programs, unless high throughput automated RT-PCR instruments are used [2]. The Center for Disease Control and Prevention has recommended that this load can be alleviated by using SARS-CoV-2 antigen tests. They suggest that those not fully vaccinated and who have not had COVID-19 in the last 3 months can be considered for antigen testing if exposed to a positive COVID-19 case within the last 14 days [3]. In long-term care facilities, antigen testing may be used for both asymptomatic and symptomatic residents/healthcare providers, as part of a COVID-19 outbreak response, or as part of expanded screening [4].

Most SARS-CoV-2 antigen tests in use are lateral flow immunoassays (LFIAs). Indeed, the WHO has included some of these under its Emergency Use Authorization List (e.g., Abbott Panbio COVID-19 antigen rapid test device, SD Biosensor Standard Q COVID-19 antigen test) [5]. However, the performance of LFIAs can vary, with one study showing an overall sensitivity of only 30.2% (95% confidence interval (CI) 21.7–39.9) for one LFIA (Coris COVID-19 Antigen Respi-Strip test) [6] in a tertiary hospital population; sensitivity improved to 70.6% when confined to those with higher viral loads (RT-PCR cycle threshold (Ct) values < 30). In another study [7], even when using a WHO-listed test (Abbott Panbio COVID-19 Antigen Rapid test), overall sensitivity was reported to be 60.8% (95% CI 46.1–74.2) among 51 RT-PCR SARS-CoV-2 positive patients; higher sensitivity (79.3%) was seen in patients with Ct ≤ 30. When four different rapid antigen tests were compared (Roche SARS-CoV-2 Rapid Antigen Test, Abbott Panbio COVID-19 Antigen Rapid Test, MEDsan SARS-CoV-2 Antigen Rapid Test, CLINITEST RAPID COVID-19 Antigen test) [8], sensitivity ranged from 44.6–54.9%. To achieve higher sensitivities (>90%), LFIAs must be confined to those with lower Ct counts. For example, the sensitivity of the Clinitest Rapid COVID-19 antigen test [9] and the Abbott Panbio COVID-19 Antigen Rapid Test [10] only increased above 90% when Ct counts were below 25. In addition, the use of rapid SARS-CoV-2 antigen tests is highly manual. Not all LFIAs can be scaled appropriately to handle large sample numbers, with few assays—such as the QIAreach SARS-CoV-2 Antigen Test (Qiagen, Hilden, Germany)—having the ability to perform more than one test at a time (8 tests simultaneously) [11]. Thus, manufacturers have developed high throughput SARS-CoV-2 antigen tests for automated platforms for the simultaneous/continuous assessment of samples. We have performed an evaluation of the new Roche Elecsys SARS-CoV-2 antigen assay, and here report our findings.

## 2. Methods

### 2.1. Study Participants

For the verification of assay sensitivity/specificity, we used archived universal transport medium samples (nasopharyngeal swabs). Archived samples had been tested for SARS-CoV-2 RT-PCR from June 2020 to June 2021. This comprised 289 RT-PCR negative cases and 61 RT-PCR positive cases. All positive samples had known RT-PCR Ct values.

This was compared to 7683 samples received for antigen testing between June to October 2021, in which RT-PCR results were available. This validation group comprised of 7657 RT-PCR negative cases and 26 RT-PCR positive cases. We excluded 9 samples from RT-PCR positive cases that were tested for SARS-CoV-2 antigen prior to RT-PCR testing.

All samples obtained in this study were unidentified and anonymous.

### 2.2. Materials and Methods

All samples were nasopharyngeal swabs collected in standard universal transport medium tubes (MANTACC UTM Transport Medium tubes, Miraclean Technology Co., Ltd., Shenzhen, China). The Roche SARS-CoV-2 antigen assay is a double-antibody sandwich electro-chemiluminescent immunoassay, where biotinylated monoclonal antibodies and ruthenylated monoclonal antibodies create a complex with nucleocapsid antigens, then binding to streptavidin-coated microparticles on a solid phase. Electrochemiluminescence is then induced by applying a voltage, with a signal yield directly proportional to the antigen titer, which is translated into a cut-off index (COI), with COI ≥ 1.0 considered positive. The assay has a claimed testing time of 18 min, and only requires 30 μL of fluid from naso-/oropharyngeal specimens placed in transport media. It has a reported inter-assay precision of 1.7–5.7%, with a limit of detection of 22.5–37.5 TCID_50_/mL (Median Tissue Culture Infectious Dose). In the manufacturer-provided information, relative sensitivity was evaluated using 232 nasopharyngeal and 158 oropharyngeal swabs. At Ct values < 26, the assay showed 100% sensitivity; however, at Ct ≤ 40, the sensitivity was 60.5%. In patients presenting ≤5 days following the onset of symptoms, sensitivity was 97.5% in those with Ct < 30, versus 26.7% in those with Ct ≥ 30. Analytical specificity, evaluated using 2747 specimens, was 99.9%. For RT-PCR analysis, samples from the verification group were analyzed on the Cobas 6800 using the Cobas SARS-CoV-2 RT-PCR assay (ORF gene and E gene), and validation cohort samples were assessed either on the Cobas 6800 (ORF gene and E gene) or the GeneXpert using Xpress SARS-CoV-2 RT-PCR assay (N gene and E gene).

Assay precision was assessed by running negative and positive controls 5 times each over 5 days. Linearity was assessed by combining a positive and negative sample to create a series of dilutions covering the clinically relevant range. We verified the analyte concentration near the cut-off that yielded 50% positive/negative results when replicates of a single sample at that concentration were tested (C_50_), and qualitative cut-off verification was performed by running two pooled serum samples that were +20% and −20% of the C_50_ five times each over 7 days [12].

### 2.3. Statistical Analysis

Data were presented in either mean ± standard deviation or median (inter-quartile range), as appropriate. No indeterminate or missing results were used. The diagnostic specificity of the test is represented by the negative percentage agreement between antigen/PCR negativity in all control subjects; diagnostic sensitivity is represented by the positive percentage agreement between antigen positivity against all RT-PCR positive patients. Confidence intervals of 95% for sensitivity and specificity were calculated according to the Clopper and Pearson exact method with standard logit confidence intervals. We also performed a concordance analysis between the antigen/PCR results and attempted to calculate the positive predictive value and negative predictive value. Receiver operating characteristic (ROC) analysis was performed between antigen COIs and Ct values of cases. All statistical analyses were performed on MedCalc Statistical Software (Version 20.008, MedCalc Software Ltd., Ostend, Belgium) with *p* < 0.05 considered statistically significant. Regression analysis was also performed for results between Ct values and antigen COIs. Our hospital’s Institutional Review Board deemed this work exempt, as this was part of routine laboratory evaluation of new assays using de-identified, anonymized samples/data. Compliance with STARD guidelines is enclosed (see Supplementary Table S1).

## 3. Results

### 3.1. Performance Evaluation

The assay had a within-run precision CV of 3.0% at COI 0.68, and CV of 1.5% at COI 3.49. Between-run precision was 3.0% at COI 0.68 and 1.8% at COI 3.49. The assay was linear from COI 0.65 to 7.84. All 35 C_50_ ± 20% test results performed over 7 days were positive/negative, respectively.

### 3.2. Sensitivity and Specificity Analysis

In the verification group, the antigen results of the 289 PCR negative cases ranged from COI 0.41–1.2, and COI 0.38–522 in the 61 RT-PCR positive cases (Cobas ORF gene Ct 16–35, E gene Ct 17–38). Out of 61 RT-PCR positive cases, 26 were antigen positive (COI 1.0–522), and 35 were negative (COI 0.381–0.995). Out of the 289 PCR negative cases, only 1 was antigen positive (COI 1.2). Overall sensitivity was thus 42.6% (95% CI 30.0–55.9), and specificity was 99.7% (95% CI 98.1–100). Positive predictive value of the assay was 96.3% (95% CI 78.2–99.5), and negative predictive value was 89.2% (95% CI 86.9–91.1).

### 3.3. ROC Analysis

**Figure 1:**
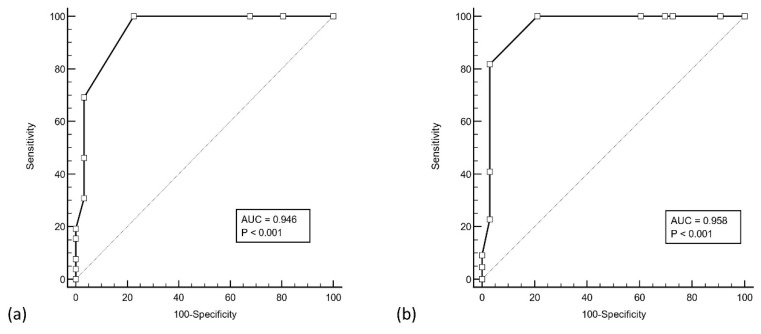
ROC analysis of Roche Cobas RT-PCR cycle threshold counts of the (**a**) ORF gene and (**b**) E gene, with antigen assay results. Both ORF and E genes yielded an associated criterion of cycle threshold count ≤ 31 with a sensitivity and specificity of 100%.

**Figure 2:**
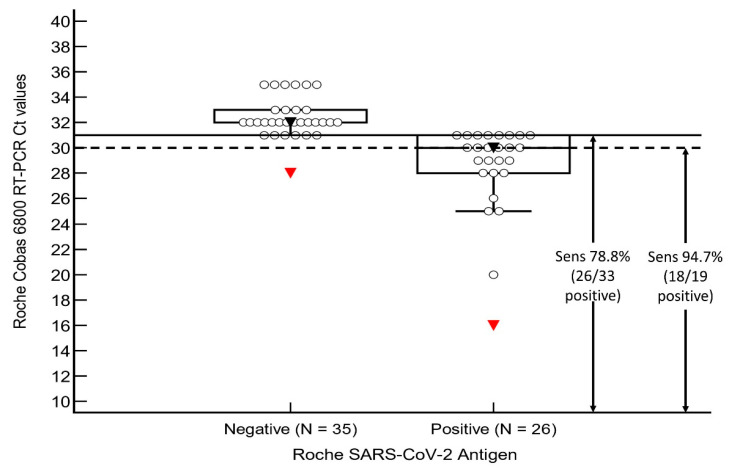
Sensitivity analysis of our validation group. Abbreviations: Ct, cycle threshold count; Sens, sensitivity.

ROC analysis of the RT-PCR ORF gene Ct counts and antigen positivity was performed in the verification group (see Figure 1). The area under curve (AUC) was 0.95 (ORF gene, 95% CI 0.85–0.99, *p* < 0.001) and 0.96 (E gene, 95% CI 0.87–0.99), with an associated criterion of Ct ≤ 31 for both genes (ORF: 100% sensitivity and specificity, E: 82% sensitivity and 100% specificity) for antigen positivity. When confined to cases with Ct counts ≤ 31, the sensitivity of the assay improved to 78.8% (26/33 positive, 95% CI 61.1–91.0) (see Figure 2), and when confined to those with Ct counts ≤ 30, the sensitivity improved even further to 94.7% (18/19 positive, 95% CI 74.0–99.9). All four cases with Ct values ≤ 25 were antigen positive (COI 5.17–522).

### 3.4. Regression Analysis

**Figure 3:**
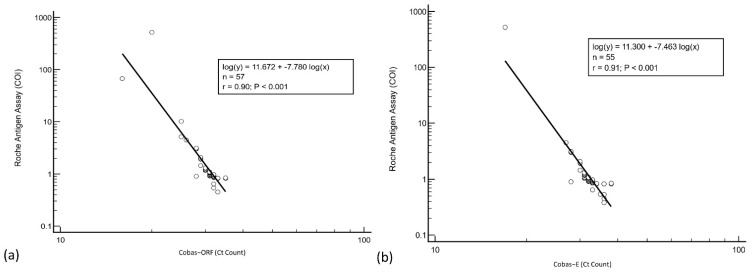
Regression analysis of Roche Cobas RT-PCR (**a**) ORF and (**b**) E gene cycle threshold counts and antigen assay cut-off index.

Regression analysis was performed to determine the agreement between RT-PCR and antigen results from the first group of cases. Overall, agreement between the two assays was good (ORF and E gene, Spearman r = 0.90) (see Figure 3).

### 3.5. Validation Group Analysis

In the validation group, the antigen results of the 7657 RT-PCR negative samples ranged from COI 0–1.33. The COI of the 17 RT-PCR positive samples ranged from COI 0.43 to 531 (Median 0.78, IQR 0.59 to 5.94). Eight of these samples were within 0–7 days after their first positive RT-PCR (COI 0.72–5314, median 9.19, IQR 0.80–121), 5 of which were reactive (COI 2.68–5314) (see Supplementary Table S2). When confined to 0–7 days after the first positive RT-PCR, positive percentage agreement was 62.5% (95% CI 24.5–91.5). However, none of the cases with an antigen test performed after the first week of infection (>7 days after the first positive RT-PCR) were reactive (COI 0.427–0.785). Thus, the antigen and RT-PCR results had a positive percentage agreement of 29.4% (95% CI 10.3–56.0) from 0–25 days after the first positive RT-PCR. Out of 7657 controls, 5 were reactive (COI 1.05–1.33), resulting in a negative percentage agreement of 99.9% (95% CI 99.85–99.98).

## 4. Discussion

The Roche Elecsys SARS-CoV-2 antigen assay demonstrates good performance, is within the manufacturer’s claims, and shows good specificity in a large control group. Although RT-PCR testing for SARS-CoV-2 is the gold standard for COVID-19 diagnosis, it is not without its flaws. One study [13] demonstrated that 4.5% (40/880) of RT-PCR negative close contacts of COVID-19 patients were positive for SARS-CoV-2 antibodies in the absence of a positive RT-PCR test. Another study [14] reported that RT-PCR can have a false-negative rate of up to 38% on the day of disease onset. By itself, RT-PCR testing protocols are not immune to missing SARS-CoV-2 infections, especially if RT-PCR tests are not readily available and cannot be performed on a frequent basis. This can cause even sensitive RT-PCR tests to miss the diagnosis of COVID-19 during the infectious window [15]. In addition, RT-PCR testing may not be readily available due to resource constraints and reagent supply chain issues. Given the good agreement between the Roche antigen assay and RT-PCR Ct counts, the Elecsys SARS-CoV-2 assay can serve as a valuable surrogate rule-in test for the diagnosis of patients with suspected COVID-19 when RT-PCR testing is not available. Other studies [16] have also demonstrated a good agreement (91.4%) between automated antigen assays (Lumipulse) and RT-PCR. Indeed, the Elecsys SARS-CoV-2 antigen assay has been compared to other antigen assays run on automated platforms (Lumipulse, LIAISON, Euroimmun), as well as the Roche rapid antigen test [17], and has shown good sensitivity in SARS-CoV-2 RT-PCR positive patients (viral loads ranging from 83 to 1,548,572,803 Geq/mL). The Roche SARS-CoV-2 antigen assay displayed a greater sensitivity than the Roche rapid antigen test (31.42% vs. 23.58%), as well as the the Euroimmun (17.76%) and LIAISON (19.63%) assays, with only the Lumipulse showing a slightly greater sensitivity (52.34%). Furthermore, an additional benefit of automated antigen tests is that they have much higher throughput than point-of-care tests, and can be rapidly scaled to handle many samples; the LIAISON antigen test has a maximal throughput of 136 tests/h [18].

In addition, the Roche antigen test showed good sensitivity in cases with Ct ≤ 30, where the sensitivity of the test exceeded 90%. We note that the Clinitest point-of-care antigen test required a Ct ≤ 25 before it could achieve sensitivity of >90% [9], whereas the Abbott Panbio Antigen Rapid Test only achieved a sensitivity of 77.8% at Ct < 30, only increasing to 97.1% at Ct < 25 [10]. Even the Abbott BinaxNOW point-of-care test showed a sensitivity of only 75.3% when restricted to subjects with Ct < 30 [19]. Indeed, compared to central laboratory tests (Lumipulse), a point-of-care LFIA (Espline SARS-CoV-2) [20] showed a sensitivity of only 41% within the first two weeks of symptom onset, versus 91% on the central assay. Thus, the improved sensitivity of central laboratory antigen tests in patients with lower viral loads (higher Ct counts) would allow for the earlier detection of COVID-19. Other recent studies also support the improved sensitivity of high-throughput antigen tests [21], with a sensitivity of 93.8–100% in subjects with Ct counts between 26–30, even across different SARS-CoV-2 variants.

However, the data from our validation group showed that the antigen test positive percentage agreement with RT-PCR results decreases rapidly after the first week of disease onset (from 62.5% to all non-reactive). Studies [22] have also demonstrated that the SARS-CoV-2 antigens are the highest at disease onset but decline quickly to sub-reactive levels after 2–3 weeks. As such, it is imperative that antigen tests be used as close to disease onset as possible, preferably within the first week of infection. This would also apply to SARS-CoV-2 antigen LFIAs. For example, the Abbott Panbio Antigen Rapid Test sensitivity was 86.5% within the first week of disease onset, compared to 53.8% thereafter [10]. The fact that 53% (9 out of 17) of our cases from the validation cohort had their first antigen tests performed only after the first week of infection shows that further education to end-users on how to properly utilize SARS-CoV-2 antigen tests is necessary. Timing is also essential, because if the initial antigen test is performed early, centralized laboratory testing can be conveniently repeated to track viral clearance in COVID-19 patients [16]. Centralized laboratory antigen test results not only closely correlate with RT-PCR results, but also demonstrate a slow declining trend from 0–20 days after symptom onset. Abrupt positive-to-negative changes are observed with RT-PCR when Ct values are not reported.

Our study reports the following:

1. The sensitivity of the Roche antigen assay can be improved when used in patients with high viral loads; sensitivity exceeds 90% in cases with Ct ≤ 30;
2. The antigen assay has a good agreement with the RT-PCR Ct values;
3. The antigen assay is most sensitive in the first week after the first positive RT-PCR.

One limitation of our study was that we were unable to compare the performance of the Roche Elecsys antigen assay to other high-throughput antigen assays. Comparison studies of different high throughput auto-analyzer SARS-CoV-2 antigen assays would be useful [17]. However, it is important to remember that SARS-CoV-2 antigen assays are not standardized, and the results of one assay may not be transferable to another. This translates into differing sensitivities across different antigen tests even among the same population, as demonstrated in the previous study that compared four different automated SARS-CoV-2 antigen platforms [17]. Another limitation of our study is the small number of RT-PCR positive cases in the validation cohort. Further studies using larger numbers of real-world subjects with parallel RT-PCR and antigen testing would be desirable.

## 5. Conclusions

In conclusion, the Elecsys SARS-CoV-2 antigen assay has good performance that is within manufacturer specifications. The sensitivity of the Roche antigen assay was greatest when used in patients with lower RT-PCR Ct values (≤30) and is best used within the first week of disease onset.

## Data Availability

Not applicable.

## Supplementary Materials

The following are available online at https://www.mdpi.com/article/10.3390/vaccines10020198/s1, Table S1: STARD checklist, Table S2: First positive RT-PCR cycle threshold counts and Antigen cut-off indexes of samples from RT-PCR positive cases in our real-world population.

## Abbreviations

RT-PCR	Reverse transcription–polymerase chain reaction
LFIA	Lateral flow immunoassay
CI	Confidence interval
Ct	Cycle threshold
COI	Cut-off index
ROC	Receiver Operating Characteristic
AUC	Area Under Curve
