# Supplementary material for: Evaluation and Validation of the Roche Elecsys SARS-CoV-2 Antigen Electro-Chemiluminescent Immunoassay in a Southeast Asian Region
Source: Vaccines (Basel). 2022 Jan 27;10(2):198. doi: 10.3390/vaccines10020198 (PMC8875833; doi:10.3390/vaccines10020198)
Supplement: Supplementary file 1 [file vaccines-10-00198-s001.zip › vaccines-1567974-supplementary.pdf]

Supplementary Table S1: STARD checklist

Section & Topic	No	Item	Reported on page #
**TITLE OR ABSTRACT**			
	**1**	Identification as a study of diagnostic accuracy using at least one measure of accuracy (such as sensitivity, specificity, predictive values, or AUC)	**2**
**ABSTRACT**			
	**2**	Structured summary of study design, methods, results, and conclusions (for specific guidance, see STARD for Abstracts)	**2**
**INTRODUCTION**			
	**3**	Scientific and clinical background, including the intended use and clinical role of the index test	**4**
	**4**	Study objectives and hypotheses	**5**
**METHODS**			
Study design	**5**	Whether data collection was planned before the index test and reference standard were performed (prospective study) or after (retrospective study)	**5**
Participants	**6**	Eligibility criteria	**5**
	**7**	On what basis potentially eligible participants were identified (such as symptoms, results from previous tests, inclusion in registry)	**5**
	**8**	Where and when potentially eligible participants were identified (setting, location and dates)	**5**
	**9**	Whether participants formed a consecutive, random or convenience series	**5**
Test methods	**10a**	Index test, in sufficient detail to allow replication	**5**
	**10b**	Reference standard, in sufficient detail to allow replication	**6**
	**11**	Rationale for choosing the reference standard (if alternatives exist)	**6**
	**12a**	Definition of and rationale for test positivity cut-offs or result categories of the index test, distinguishing pre-specified from exploratory	**6**
	**12b**	Definition of and rationale for test positivity cut-offs or result categories of the reference standard, distinguishing pre-specified from exploratory	**6**
	**13a**	Whether clinical information and reference standard results were available to the performers/readers of the index test	**6**
	**13b**	Whether clinical information and index test results were available to the assessors of the reference standard	**6**
Analysis	**14**	Methods for estimating or comparing measures of diagnostic accuracy	**6**
	**15**	How indeterminate index test or reference standard results were handled	**6**

	**16**	How missing data on the index test and reference standard were handled	**6**
	**17**	Any analyses of variability in diagnostic accuracy, distinguishing pre-specified from exploratory	**7**
	**18**	Intended sample size and how it was determined	**Not applicable**
**RESULTS**			
Participants	**19**	Flow of participants, using a diagram	**Not applicable**
	**20**	Baseline demographic and clinical characteristics of participants	**5**
	**21a**	Distribution of severity of disease in those with the target condition	**Not applicable**
	**21b**	Distribution of alternative diagnoses in those without the target condition	**Not applicable**
	**22**	Time interval and any clinical interventions between index test and reference standard	**Not applicable**
Test results	**23**	Cross tabulation of the index test results (or their distribution) by the results of the reference standard	**7**
	**24**	Estimates of diagnostic accuracy and their precision (such as 95% confidence intervals)	**Not applicable**
	**25**	Any adverse events from performing the index test or the reference standard	**Not applicable**
**DISCUSSION**			
	**26**	Study limitations, including sources of potential bias, statistical uncertainty, and generalisability	**11**
	**27**	Implications for practice, including the intended use and clinical role of the index test	**11**
**OTHER INFORMATION**			
	**28**	Registration number and name of registry	**7**
	**29**	Where the full study protocol can be accessed	**Not applicable**
	**30**	Sources of funding and other support; role of funders	**Not applicable**

Supplementary Table S2: First positive RT-PCR cycle threshold counts and Antigen cut-off indexes of samples from RT-PCR positive cases in our real-world population.

Patient	Collection date	Roche RT-PCR (Ct Count)		GeneXpert RT-PCR (Ct Count)		SARS-CoV-2 Antigen (COI) *	Days post-first positive RT-PCR
		ORF gene	E gene	N gene	E gene		
**Negative for SARS-CoV-2 Antigen**							
A	4/8/2021	33.27	35.43				
	21/8/2021					< 1.0	17
	22/8/2021					< 1.0	18
	25/8/2021					< 1.0	21
	29/8/2021					< 1.0	25
B	14/8/2021	34.28	37.03				
	25/8/2021					< 1.0	9
C	1/9/2021	23.31	23.76				
	25/9/2021					< 1.0	24
D	20/9/2021					< 1.0	0
	20/9/2021			33	31		
E	3/9/2021	27.73	28.84				
	17/9/2021					< 1.0	14
	27/9/2021					< 1.0	24
F	8/9/2021			35	35		
	15/9/2021					< 1.0	7
G	8/9/2021			21	18		

	26/9/2021					< 1.0	18
**Positive for SARS-CoV-2 Antigen**							
H	26/9/2021	23.53	23.87				
	28/9/2021					15.7	2
I	23/9/2021			19	16		
	27/9/2021					208	4
J	20/9/2021			24	21		
	20/9/2021					34.7	0
K	20/9/2021	27.64	28.01				
	20/9/2021					< 1.0	0
	27/9/2021					5314	7
L	26/9/2021	28.77	29.43				
	28/9/2021					2.68	2

***** *- The Cut-off Index of all Negative antigen tests are <1.0 and exact values not displayed.*

Abbreviations: Ct: Cycle Threshold Count, COI: Cut-off index
